# Surface‒Aerosol Stability and Pathogenicity of Diverse Middle East Respiratory Syndrome Coronavirus Strains, 2012‒2018

**DOI:** 10.3201/eid2712.210344

**Published:** 2021-12

**Authors:** Neeltje van Doremalen, Michael Letko, Robert J. Fischer, Trenton Bushmaker, Jonathan Schulz, Claude K. Yinda, Stephanie N. Seifert, Nam Joong Kim, Maged G. Hemida, Ghazi Kayali, Wan Beom Park, Ranawaka A.P.M. Perera, Azaibi Tamin, Natalie J. Thornburg, Suxiang Tong, Krista Queen, Maria D. van Kerkhove, Young Ki Choi, Myoung-don Oh, Abdullah M. Assiri, Malik Peiris, Susan I. Gerber, Vincent J. Munster

**Affiliations:** National Institutes of Health, Hamilton, Montana, USA (N. van Doremalen, M. Letko, R.J. Fischer, T. Bushmaker, J. Schulz, C.K. Yinda, S.N. Seifert, V.J. Munster);; Washington State University, Pullman, Washington, USA (M. Leitko, S.N. Seifert);; Seoul National University College of Medicine, Seoul, South Korea (N.J. Kim, W.B. Park, M.-d. Oh);; King Faisal University, Al-Hasa, Saudi Arabia (M.G. Hemida);; Kafrelsheikh University, Kafrelsheikh, Egypt (M.G. Hemida);; University of Texas Health Sciences Center, Houston, Texas, USA (G. Kayali);; University of Hong Kong, Hong Kong, China (R.A.P.M. Perera, M. Peiris);; Centers for Disease Control and Prevention, Atlanta, Georgia, USA (A. Tamin, N.J. Thornburg, S. Tong, K. Queen, S.I. Gerber);; World Health Organization, Geneva, Switzerland (M.D. van Kerkhove);; Chungbuk National University, Cheongju City, South Korea (Y.K. Choi); Ministry of Health, Riyadh, Saudi Arabia (A.M. Assiri)

**Keywords:** Middle East respiratory syndrome coronavirus, MERS-CoV, coronavirus, viruses, severe acute respiratory syndrome coronavirus 2, SARS-CoV-2, respiratory infections, surface‒aerosol stability, pathogenicity, strains, respiratory infections, zoonoses

## Abstract

Middle East respiratory syndrome coronavirus (MERS-CoV) infects humans and dromedary camels and is responsible for an ongoing outbreak of severe respiratory illness in humans in the Middle East. Although some mutations found in camel-derived MERS-CoV strains have been characterized, most natural variation found across MERS-CoV isolates remains unstudied. We report on the environmental stability, replication kinetics, and pathogenicity of several diverse isolates of MERS-CoV, as well as isolates of severe acute respiratory syndrome coronavirus 2, to serve as a basis of comparison with other stability studies. Although most MERS-CoV isolates had similar stability and pathogenicity in our experiments, the camel-derived isolate C/KSA/13 had reduced surface stability, and another camel isolate, C/BF/15, had reduced pathogenicity in a small animal model. These results suggest that although betacoronaviruses might have similar environmental stability profiles, individual variation can influence this phenotype, underscoring the need for continual global viral surveillance.

Middle East respiratory syndrome coronavirus (MERS-CoV) was detected during 2012 and continues to cause outbreaks as a result of frequent spillover from dromedary camels to humans. Human infection with MERS-CoV has a mortality rate of ≈35%, and the virus has spread to 27 countries (*1*). Approximately 41% of human MERS-CoV infections in Saudi Arabia are primary, resulting from direct camel-to-human transmission (*2*). To date, MERS-CoV has been detected in camels in Burkina Faso, Egypt, Ethiopia, Jordan, Kenya, Morocco, Nigeria, Saudi Arabia, Senegal, Sudan, Tunisia, and Uganda (*3*–*11*).

Human-to-human transmission of MERS-CoV primarily occurs in hospital settings and within households (*12*). Epidemiologic studies have mapped indirect patient contact within hospitals, providing evidence for aerosol-mediated and hospital-worker–mediated spread (*13*–*16*). The largest outbreak of infection with MERS-CoV outside the Middle East occurred when 1 traveler from the Middle East brought MERS-CoV to South Korea, resulting in 185 subsequent infections (*17*).

Coronaviruses have large, nonsegmented, positive-sense RNA genomes. The 1% nucleotide sequence variation reported between various MERS-CoV isolates collected in the Middle East and North Africa is equivalent to 300 nt changes in the 30-kB viral genome (*18*). Many of these changes are nonsynonymous and distributed throughout the viral genome. Even single amino acid changes in MERS-CoV can alter viral replication (*19*), and deletions in MERS-CoV have been shown to attenuate pathology in an animal model (*18*). These findings underscore the need for characterizing how MERS-CoV genetic variation alters viral replication, pathogenicity, and stability.

We tested a broad panel of viral isolates collected from humans and camels, representing every major geographic region that has had MERS-CoV outbreaks and spanning from early to contemporary outbreaks. Because MERS-CoV spreads within households and hospitals, we characterized viral phenotypes with immediate implications for public health. We focused on environmental stability in aerosols as well as surface stability on common materials found in hospitals, replication kinetics in immortalized human cell lines and primary human airway epithelial cultures, and pathogenicity in a transgenic mouse model our laboratory developed to test vaccine efficacy (*20*). For environmental stability studies, we included severe acute respiratory syndrome coronavirus 2 (SARS-CoV-2) to enable better comparison of these findings with those of previously published stability studies (*21*).

## Methods

### Ethics

Animal experiment approval was obtained by the Institutional Animal Care and Use Committee at Rocky Mountain Laboratories, National Institutes of Health (Hamilton, MT, USA). All animal experiments were executed in an Association for Assessment and Accreditation of Laboratory Animal Care‒approved facility, following the guidelines in National Institutes of Health Guide for the Care and Use of Laboratory Animals, Animal Welfare Act, US Department of Agriculture, and United States Public Health Service Policy on Humane Care and Use of Laboratory Animals. The Institutional Biosafety Committee approved work with MERS-CoV strains under Biosafety Level 3 conditions.

### Viral Stock Propagation

We provide strain-specific details for the viruses used in this study (Table). Viruses were isolated by others and provided for this study. SARS-CoV-2/Washington was isolated by the Centers for Disease Control and Prevention (Atlanta, GA, USA).

**Table:** Characteristics of Middle East respiratory syndrome coronaviruses tested*

Name	Host	Year	Location	Full name	GenBank accession no.	SNPs >50%
EMC/12	Human	2012	Saudi Arabia	HCoV-EMC/2012	JX869059	G27162A (ORF5, W108†)
U/14	Human	2014	United States	Hu/Florida/USA-2/Saudi Arabia/2014	KP223131	None
KSA/15	Human	2015	Saudi Arabia	Hu/Hofuf/KSA-11002/2015	KY688120	None
SK/15	Human	2015	South Korea	Hu/Korea/Seoul/177–3/2015	KX034100	C2149A (NSP2, S431Y); A6884G (synonymous); T9566C (synonymous); G10155T (NSP5, A46S); A11376T (NSP6, S147C); C14162T (synonymous); C26189T (ORF4b R33C)
KSA/18	Human	2018	Saudi Arabia	Hu/Saudi Arabia/3015600912/2018	MN723544	C21149A (NSP16, L183I); G22366A† (S, R304Q); C25009T (S, S1185L)
C/KSA/13	Camel	2013	Saudi Arabia	Camel/Saudi Arabia/KFU-HKU1/2013	KJ650297	C25207T (S, S1251F); C27875T (M, T8I)
C/E/13	Camel	2013	Egypt	Camel/Egypt/NRCE/HKU270/2013	KJ477103	T16318C (synonymous); C24112T (S, A886V); 26892T (ORF5, P18L)
C/BF/15	Camel	2015	Burkina Faso	Camel/Burkina Faso/CIRAD-HKU785/2015	MG923471	None

*NSP, nonstructural protein; ORF, open reading frame; SNP, single-nucleotide polymorphism. †Present in other sequences.

We obtained MERS-CoV strains from the following sources: EMC12 from Erasmus Medical Center (Rotterdam, the Netherlands); U/14, KSA/15, and KSA/18 from the Centers for Disease Control and Prevention; SK/15 from Chungbuk National University (Cheongju, South Korea); and C/KSA/13, C/E/13, and C/BF/15 from Hong Kong University (Hong Kong, China). We passaged MERS-CoV and SARS-CoV-2 strains once in Vero E6 cells in Dulbecco’s modified Eagle medium (DMEM; Sigma Aldrich, https://www.sigmaaldrich.com) supplemented with 2% fetal bovine serum (Thermo Fisher Scientific, https://www.thermofisher.com), 50 U/mL of penicillin (Thermo Fisher), and 50 μg/m of streptomycin (Thermo Fisher). 

We maintained Vero E6 cells in DMEM supplemented with 10% fetal bovine serum, 1 mmol/L of l-glutamine, 50 U/mL of penicillin, and 50 μg/mL of streptomycin. We clarified virus stocks by centrifugation and froze them at −80°C. We performed virus titrations by using endpoint titration in Vero E6 cells inoculated with 10-fold serial dilutions of virus. We scored cytopathic effect at day 5 (for MERS-CoV) or day 6 (for SARS-CoV-2) and calculated median tissue culture infectious dose (TCID_50_) from 4 replicates by using the Spearman‒Karber method (*22*).

### Sequencing Stocks

We treated MERS-CoV samples with RiboZero H/M/R rRNA Depletion Mix (Illumina, https://www.illumina.com) according to the manufacturer’s instructions. After purification with Ampure RNACleanXP (Beckman Coulter, ttps://www.beckmancoulter.com), we eluted enriched RNA and assessed it on a BioAnalyzer RNA Pico Chip (Agilent Technologies, https://www.agilent.com). We prepared second-strand cDNA according to the Truseq Stranded mRNA Library Preparation Guide (Illumina). We treated samples with RiboShredder RNase Blend (https://www.cambio.co.uk).

We visualized final libraries on a BioAnalyzer DNA1000 Chip (Agilent Technologies), and quantified them by using a KAPA Library Quant Kit (Illumina) and a universal qPCR Mix (Kapa Biosystems, https://www.roche.com) on a CFX96 Real-Time System (Bio-Rad Laboratories, https://www.bio-rad.com). We pooled libraries together in equimolar concentrations and sequenced them using MiSeq (Illumina) with on-board cluster generation and 2 × 250 paired-end sequencing. The cluster density was at 454 k/mm^2^/lane, resulting in 8.7 million reads passing filter/run and an average 85% greater than the Q30 score.

### Phylogenetics

We downloaded all available MERS-CoV genome sequences from GenBank and curated them to remove sequences that were not independently sampled. We aligned sequences with the consensus sequences for MERS-CoV isolates used in this study by using MAFFT version 7.388 plugin (*23*) in Geneious Prime (https://www.geneious.com). We inferred a phylogenetic tree by using the maximum-likelihood method under the general time reversible plus gamma model of nucleotide substitution with 1,000 bootstrap replicates implemented with PhyML version 3.3.20190321 (https://www.atgc-montpellier.fr).

### Stability of MERS-CoV on Surfaces and in Aerosols

We sterilized 15-mm polypropylene discs (ePlastics, https://www.eplastics), AISI 304 alloy stainless steel discs (Metal Remnants, https://metalremnants.com), copper discs (99.9%; Metal Remnants), and silver discs (99.9%) (Sigma-Aldrich, https://www.sigmaaldrich.com), placed them in 24-well plates, and added 50 μL of MERS-CoV (10^5^ TCID_50_/mL). For timepoints taken at 0, 1, 24, 48, and 72 h, we added 1 mL of DMEM to wells, aliquoted, and stored at −80°C. We titrated samples on Vero E6 cells and maintained the temperature (21°C–22°C) and humidity (45%–55%).

We determined virus stability in aerosols as described (*24*). In brief, we loaded a collison nebulizer with 10^6.5^ TCID_50_/mL of MERS-CoV in DMEM containing 2% fetal bovine serum. Aerosols were maintained in a Goldberg drum and samples collected at 0, 30, 60, 120, and 180 min after aerosolization by passing air at a volume of 6 L/min for 30 s from the drum through a 47-mm gelatin filter (Sartorius, https://www.sartorius.com). Filters were dissolved in 10 mL of DMEM containing 10% fetal bovine serum and stored at −80°C. All samples were titrated on Vero E6 cells.

### Replication of MERS-CoV Strains In Vitro

We inoculated Vero E6 cells with virus (multiplicity of infection = 0.01) and collected supernatants at 8, 24, 48 and 72 hours postinfection (hpi). Human airway epithelium (HAE) inserts (Epithelix, https://www.epithelix.com) were maintained as specified by the manufacturer. We washed HAEs with 200 μL of phosphate-buffered saline for 30 min, followed by inoculation with MERS-CoV at a multiplicity of infection of 0.1. We obtained samples at 8, 24, 48, 72, and 96 hpi.

### Animal Experiments

We inoculated intranasally transgenic BALB/c mice expressing human DPP4 with 10^3^ TCID_50_ MERS-CoV. Mice were weighed and swabbed daily. At day 3, we euthanized 4 mice and harvested lung tissue. We monitored the remaining 6 mice for survival. We euthanized mice if there were signs of severe disease signs based on quantitative assessment (e.g., hunched posture, lack of movement) or >20% weight loss.

### RNA Extraction and Quantitative Reverse Transcription PCR

We homogenized lung tissues and extracted RNA by using the RNeasy method (QIAGEN, https://www.qiagen.com) according to the manufacturer’s instructions. We added swab specimens to 1 mL of DMEM, vortexed them, and used 140 μL for RNA extraction by using the QiaAmp Viral RNA Kit and a QIAxtractor (QIAGEN).

We detected MERS-CoV viral RNA by using the UpE MERS-CoV assay (*25*) and the Rotor-GeneTM Probe Kit (QIAGEN). Primers in this assay target a highly conserved region upstream of MERS-CoV envelope gene. Sequences of MERS-CoV strains used in this study are identical in this region. MERS-CoV dilutions with known genome copies were run in parallel to enable calculation of genome copies in samples.

### Histologic and Immunohistochemical Analysis

We fixed harvested tissues for >7 days in 10% neutral-buffered formalin, processed them by using a VIP-6 Tissue Tek Tissue Processor (Sakura Finetek, https://www.sakuraus.com), and embedded them in Ultraffin Paraffin Polymer (Cancer Diagnostics, https://www.cancerdiagnostics.com). We stained 5-μm sections with hematoxylin and eosin and detected coronavirus immunoreactivity by using MERS-CoV nucleocapsid protein rabbit antibody (diluted 1:4,000; Sino Biological Inc, https://www.sinobiological.com).

We processed tissues for immunohistochemical analysis by using the Discovery ULTRA Automated IHC/ISH Staining Instrument (and a Discovery ChromoMap DAB Kit (both from Ventana Medical Systems, https://diagnostics.roche.com). For morphometric analysis, we scanned slides by using the Aperio ScanScope AT2 (Aperio Technologies, Inc., https://www.aperio.com) and analyzed the entire section by using ImageScope Positive Pixel Count Algorithm version 9.1 (Aperio Technologies, Inc.). All tissue slides were evaluated by a board-certified veterinary anatomic pathologist.

### Statistical Analyses

We performed analyses by using GraphPad Prism version 7.05 for Windows (https://www.graphpad.com). All strains were compared with EMC/12. For aerosol stability data analysis, we determined linear regression for the mean value of 3 runs/virus. We determined statistical significance in deviation from MERS-CoV/EMC12 results by using 1-way analysis of variance, followed by the Bonferroni multiple comparisons test or a 2-way unpaired Student’s t-test. We used simple linear regression to evaluate slopes of decay. Survival of mice compared with mice inoculated with EMC/12 was performed by using the log-rank (Mantel-Cox) test. To calculate the amount of virus shedding per mouse in in vivo comparisons, we calculated the area under the curve for a plot of the viral load measured in oropharyngeal swab specimens.

## Results

### Stability of MERS-CoV Strains in Aerosols or as Fomites Compared with SARS-CoV-2

**Figure 1:**
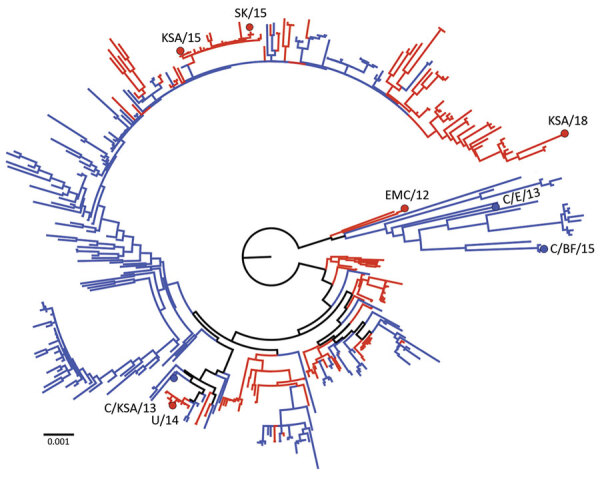
Phylogenetic tree of 446 full Middle East respiratory syndrome coronavirus (MERS-CoV) genomes showing distribution of human-derived (red) and camel-derived (blue) isolates. The tree was constructed with PhyML (https://www.atgc-montpellier.fr) and rooted at the midpoint. Strain EMC/12 was obtained from Erasmus Medical Center (Rotterdam, the Netherlands); U/14, KSA/15, and KSA/18 from the Centers for Disease Control and Prevention (Atlanta, GA, USA); SK/15 from Chungbuk National University (Cheongju, South Korea); and C/KSA/13, C/E/13, and C/BF/15 from Hong Kong University (Hong Kong, China). Scale bar indicates nucleotide substitutions per site. KSA, Kingdom of Saudi Arabia.

We selected 8 MERS-CoV strains and 1 SARS-CoV-2 strain (SARS-CoV-2/WA1-2020) to be used in this study (Table; Figure 1). Five MERS-CoV strains were isolated from human cases and 3 strains were isolated from dromedary camels. Strains were isolated during 2012‒2018 and originated from the Middle East (*5*), Africa (*2*) or South Korea (*1*) (Table). All originally obtained viruses were passaged once in Vero E6 cells, and virus stocks were deep sequenced (Table). We used MERS-CoV sequences to construct a phylogenetic maximum-likelihood tree, which showed a wide distribution of MERS-CoV strains selected. Thus, our panel represents a broad sample of known genetic variation within currently circulating MERS-CoV strains.

**Figure 2:**
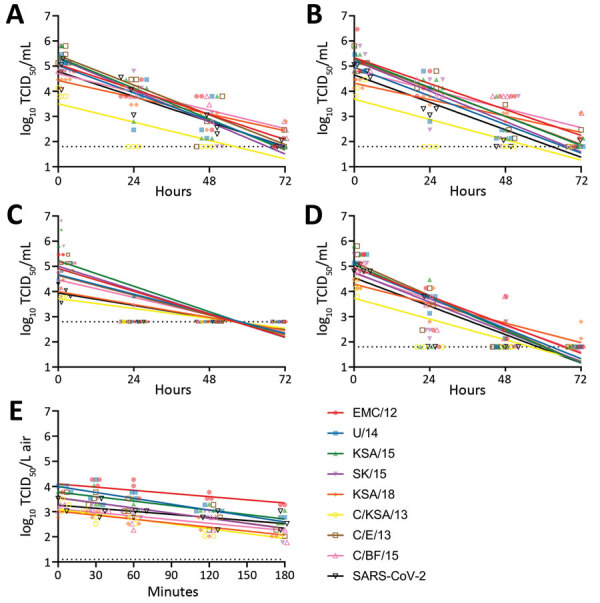
Stability of MERS-CoV strains on surfaces and in aerosols compared with those for SARS-CoV-2. Simple linear regression of virus was used for different surfaces and in aerosols. For surface stability, 50 μL of MERS-CoV or SARS-CoV-2 was spread on a surface, either polypropylene, stainless steel, copper, or silver; 1 mL of Dulbecco’s modified Eagle medium was added at times 0, 1, 24, 48, or 72 hours, and samples were titrated. For aerosol stability, MERS-CoV‒ or SARS-CoV-2‒containing aerosols were sprayed into a Goldberg drum; samples were taken at times 0, 30, 60, 120, and 180 min and then titrated. Linear regression was calculated per virus and indicated as lines. Dotted lines indicate limits of detection. Strain sources are listed in the legend for Figure 1. MERS-CoV, Middle East respiratory syndrome coronavirus; SARS-CoV-2, severe acute respiratory syndrome coronavirus 2; TCID_50_, median tissue culture infectious dose.

We first investigated the stability of MERS-CoV as fomites on polypropylene, stainless-steel, copper, and silver surfaces, which we selected because they represent commonly encountered surfaces in hospital environments or have virocidal properties (*23*). For comparison with a pandemic human coronavirus, we also included SARS-CoV-2. Back-titrations of all virus strains showed comparable starting virus titers. Stability of MERS-CoV on polypropylene and stainless-steel surfaces, maintained at 21°C–22°C and a relative humidity of 45%–55% under standard laboratory light conditions, was similar to that reported for MERS-CoV and SARS-CoV-2 stability on surfaces (*21*,*26*). We found major differences in decay rates when comparing EMC/12 to SK/15, KSA/18, C/KSA/13, and C/BF/15 on polypropylene. These differences were not found for the other surfaces (Figure 2; Appendix Figure). Infectious virus titers were low for all strains on copper and silver surfaces at 24 hours. We analyzed data by using linear regression for the first 24 hours for each surface and each virus. Decay, averaged between all virus strains, was higher for copper (−0.11576 log_10_ TCID_50_/h) and silver (−0.08744 log_10_ TCID_50_/h) surfaces than for polypropylene (−0.0529 log_10_ TCID_50_/h) and stainless-steel (−0.0469 log_10_ TCID_50_/h) surfaces.

We aerosolized all MERS-CoV strains in a Goldberg drum at 21°C and a relative humidity of 60%–70% in the dark. We then tested samples at 0, 30, 60, 120 and 180 min after aerosolization, titrated them, and compared results with those for SARS-CoV-2. We detected no major differences in linear regression of loss of infectious virus in aerosols between strains. For all MERS-CoV strains, infectious virus could still be detected at 180 min after aerosolization (Figure 2, panel B).

### In Vitro Replication of MERS-CoV Strains

**Figure 3:**
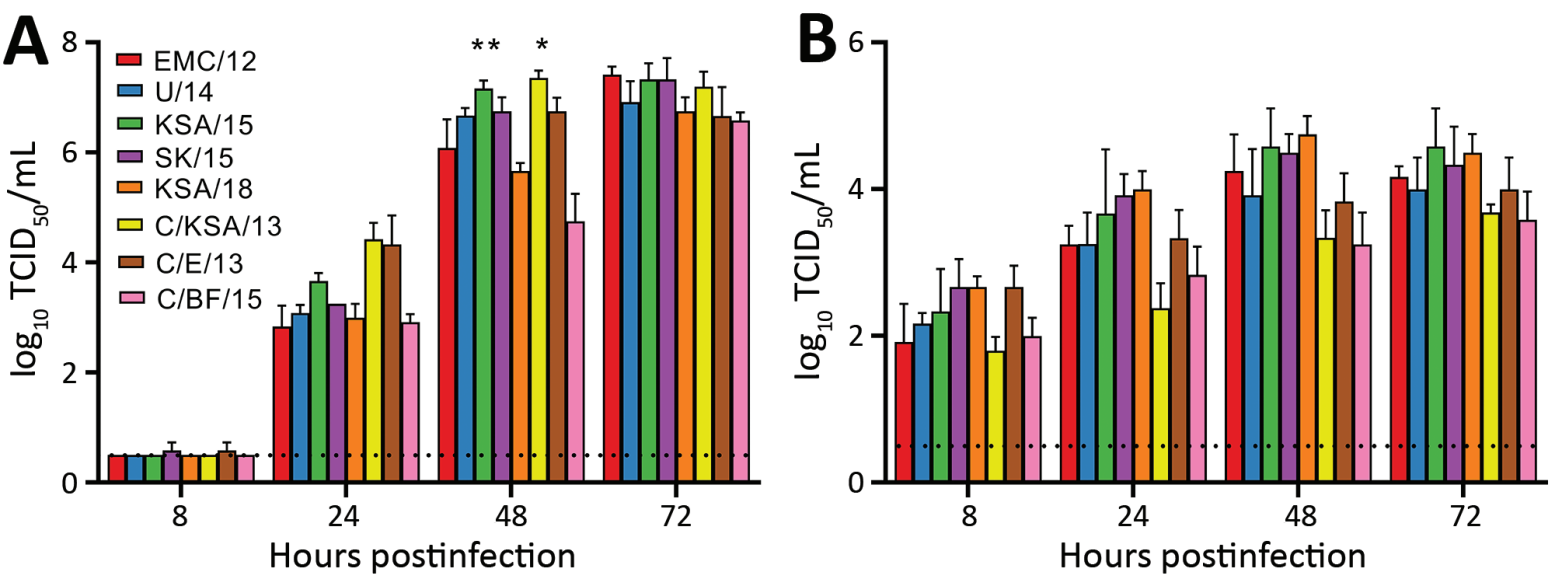
Middle East respiratory syndrome coronavirus replication in Vero E6 cells (A) and human airway epithelium (B). Replication is shown as geometric means; error bars indicate SDs. Vero E6 cells were infected with a multiplicity of infection of 0.01, and human airway epithelium were infected with a multiplicity of infection of 0.1. Samples of supernatants were obtained at 8, 24, 48 and 72 hours postinoculation and titrated. Statistically significant differences compared with those for the prototypical strain, EMC/12, were calculated by using ordinary 1-way analysis of variance, followed by a Bonferroni multiple comparisons test. Dotted lines indicate limits of detection. Strain sources are listed in the legend for Figure 1. TCID_50_, median tissue culture infectious dose. *p<0.05; **p<0.01.

To investigate any in vitro growth differences, we grew strains in 2 cell systems, Vero E6 cells and HAE cultures, in comparison to the reference strain EMC/12. At 48 hpi, C/KSA/13 and KSA/15 showed higher titers than EMC/12 in Vero E6 cells. At 72 hpi, C/KSA/13 and C/BF/15 showed lower titers than EMC/12 in HAE cultures. We observed no other major differences in either culture type. Although differences were not always statistically significant, all camel-derived viruses had reduced replication kinetics compared with those for EMC/12 in HAE cells at 24–72 hpi (Figure 3).

### Disease Progression for MERS-CoV Strains in hDPP4 Transgenic Mice

**Figure 4:**
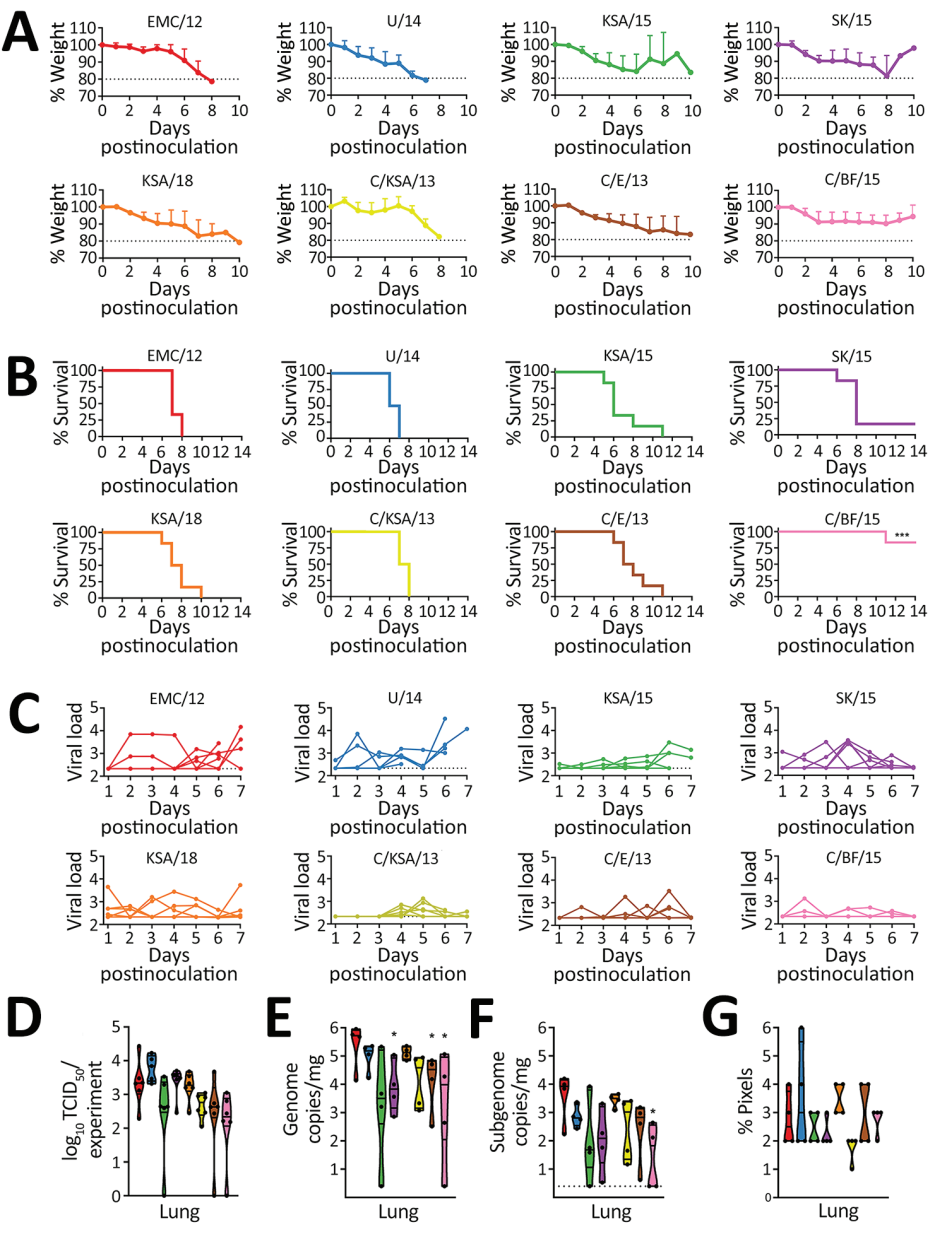
In vivo replication of different Middle East respiratory syndrome coronavirus (MERS-CoV) strains. hDPP4 mice were inoculated intranasally with 10^3^ TCID_50_ of MERS-CoV. Four mice were euthanized on day 3, and the remaining 6 mice were monitored for survival. A) Relative weight loss of hDPP4 mice. B) Survival of hDPP4 mice. C) Oropharyngeal shedding of MERS-CoV as measured by using an UpE quantitative reverse transcription PCR. D) Amount of shedding per experiment per mouse calculated by using area under the curve (AUC) analysis of viral load in oropharyngeal swab specimens. Results are displayed per mouse per virus strain. E) Viral load in lung tissue obtained from mice euthanized at day 3. F) Viral mRNA load in lung tissue obtained from mice euthanized at day 3. G) Percentage of positive pixels quantified from lung tissues stained for MERS-CoV antigen. Colors in panels D‒F match those for strains in panels A‒-C; strain sources are listed in the legend for Figure 1. Statistical significance was compared by using 1-way analysis of variance, followed by a Bonferroni multiple comparisons test. Dotted lines indicate limits of detection. TCID_50_, median tissue culture infectious dose. *p<0.05.

MERS-CoV enters cells expressing the receptor human dipeptidyl peptidase IV (hDPP4). Our laboratory developed hDPP4 transgenic mice to test MERS-CoV vaccine efficacy (*20*). We intranasally inoculated 10 mice/group with 10^3^ TCID_50_ MERS-CoV/mouse. Mice started to lose weight on days 2‒5 postchallenge; weight continued to decrease for all groups, except for mice inoculated with C/BF/15, in which only 1 mouse continued to lose weight (Figure 4, panel A). For all groups, including C/BF/15, weight loss was also associated with other signs: ruffled coat, increased breathing rate, reluctance to move, and hunched posture. Only animals in the groups inoculated with SK/15 (1/6) and the group inoculated with C/BF/15 (5/6) survived. Average time to death was similar for all groups, excluding C/BF/15: EMC/12, 7.33 days; U/14, 6.5 days; KSA/15. 7 days; SK/15. 7.6 days; KSA/18. 7.67 days; C/KSA/13, 7.5 days; and C/E/13, 8 days (Figure 4, panel B).

We measured viral RNA in oral swab specimens obtained during days 1–7 postchallenge and found no major differences in the amount of shedding between different groups (Figure 4, panels C, D). Viral genome RNA was lower in lung tissue collected on day 3 from mice inoculated with SK/15, C/E/15, and C/BF/15. Subgenomic RNA was lower to a major degree only in lung tissue of mice inoculated with C/BF/15 (Figure 4, panels E, F).

We observed no differences in pathology between different groups. Animals rarely showed pulmonary pathology at day 3. However, animals that had lesions showed only a minimal and random lymphocytic infiltrate. Immunohistochemical analysis showed that MERS-CoV antigen was expressed rarely or randomly in type I and II pneumocytes and not located in areas of inflammation. Morphometric analysis of pulmonary tissue that had immunoreactivity showed no major differences between groups (Figure 4, panel G).

## Discussion

The ongoing MERS-CoV endemic in the Middle East and subsequent discovery of the virus in camel herds across Africa has resulted in a wealth of publicly available genetic data for various viral strains and isolates. In this study, we assessed several of these isolates for viral phenotypes related to public health in an attempt to better inform public health policy making with regards to MERS-CoV and other human coronaviruses that cause respiratory diseases, such as SARS-CoV-2.

Because nosocomial spread is at the center of MERS-CoV outbreaks, we assessed the stability of the virus on various surface material types commonly found in hospitals (polypropylene plastic and stainless steel), as well as materials that had potential antiviral and known antimicrobial properties (silver and copper) (*27*,*28*). Our experiments were performed at environmental conditions similar to those in hospitals, in which there is high risk for human-to-human, nosocomial transmission. Regardless of the surface material tested, strain C/KSA/13 was the least stable over time and was below detectable levels by 24 hours (Figures 2, 4). This strain had the lowest starting titer in these experiments, which might explain this difference in stability. In addition, our C/KSA/13 stock contains 2 nonsynonymous mutations in the viral structural proteins, spike and matrix, not found in our other strains, which might also play a role in this difference, either directly or indirectly (Table). These findings warrant further studies on how specific MERS-CoV polymorphisms in structural proteins affect viral growth.

As shown by Doremalen et al. (*21*), all virus strains tested had notably reduced stability on copper and silver surfaces (Figure 2, panel A). Copper has been shown to also have antiviral properties against influenza A(H1N1) virus and SARS-CoV-2 (*29**‒**31*). The exact antiviral mechanism for copper is still unclear, but might be related to formation of hydroxyl radicals by copper ions when in aqueous solution (*31*). Silver-based nanoparticles have been shown to be antiviral for HIV-1 (*32*), herpes simplex virus 2 (*33*), hepatitis B virus (*34*), respiratory syncytial virus (*35*), and monkeypox virus (*36*). Taking advantage of the antiviral properties of copper and silver might help decrease nosocomial transmission. Both silver and copper can be used for coating medical tools (*37*) and commonly touched items, such as bed rails, door handles, and intravenous poles (*38*). These findings appear to be more broadly applicable for other coronaviruses because we observed similar results for SARS-CoV-2 (Figure 2) (*21*). Further research should be invested in determining coronavirus susceptibility to metal ion inactivation.

MERS-CoV transmission might occur through aerosols and fomites (*39*), although the role of each route is not known. Transmission often occurs in hospitals; thus, aerosol-generating medical procedures might play a major role (*40*). MERS-CoV transmission has occurred over distances of >6 feet (*41*), and evidence of MERS-CoV on surfaces and in air in hospitals has been found (*39*). Studies have suggested that a hospital air-handling system might have contributed to nosocomial spread during the 2015 MERS-CoV outbreak in South Korea (*14*,*39*), and our group has shown that the virus can remain viable suspended in air for <10 min (*26*). We tested aerosol stability of viral isolates and observed that all viruses remained viable for a minimum of 180 min with an ≈10-fold reduction in viral titer observed on average within the collected aerosols (Figure 2, panel B).

Although we did not observe major differences in this study, strain stability is an useful phenotype to continue monitoring because mutations in viral capsid proteins have been shown to enhance environmental stability of bacteriophages, dengue virus, and transmissible gastroenteritis virus (*42*–*44*). Because MERS-CoV isolates contain polymorphisms throughout the entire viral genome, including the structural proteins that form virions, mutations might arise that influence overall virus particle stability. C/KSA/13, which showed reduced stability on surfaces in our experiments, contains polymorphisms in open reading frame 1b, the spike glycoprotein, and the virion matrix protein in comparison to the other strains tested. Recent studies have further demonstrated the influence of various external factors on environmental stability for SARS-CoV-2, including experimental ambient conditions and matrix in which the virus is suspended (*45*,*46*). Our experiments were performed in standard, indoor laboratory settings and with virus suspended in culture media, which enabled us to observe intrinsic differences determined solely at the viral level. Tracking and assessing the stability of coronavirus strains will improve our understanding of coronavirus variant spread.

We tested viral replication kinetics in Vero E6 cells and primary HAE cells (Figure 3). All viruses replicated to similar titers on Vero E6 cells by 72 hours. However, KSA/15 and C/KSA/13 had higher titers than EMC/12 by 48 hpi. Albeit the difference is not significant, C/BF/15 has a lower viral titer than EMC/12 at 48 hpi and 72 hpi. These results are consistent with those of a previous study, which showed that C/BF/15 has impaired replication (*18*). In primary HAE cultures, all camel-derived viral isolates had reduced replication kinetics compared with that for EMC/12 (Figure 3, panel B). More studies are needed with these camel-derived isolates to determine whether their differences in replication kinetics results from a comparison with EMC12, which has well-described tissue culture adaptations, or to see if MERS-CoV might adapt in humans after transmission from camels. Sequence analysis of the viral variants did not identify any obvious mutation patterns in any single viral protein that would explain the differences in replication kinetics. Thus, we speculate that these differences are the result of cumulative effects across >1 types of genetic variation.

We have shown that MERS-CoV replicates in type I and II pneumocytes in the lower respiratory tract of an animal model (*20*). Although disease progression after infection with this virus does not involve the central nervous system in humans, this small animal model is suitable for vaccine candidate testing, using animal survival or viral-induced death as a binary readout for vaccine efficacy. MERS-CoV C/BF/15 contains a deletion in open reading frame 4b, which has been shown in a similar mouse model to result in impaired suppression of the host interferon response and increased type I and type III interferon signaling (*18*). Taken together, these results pave the way for testing MERS-CoV vaccine candidates for broadly neutralizing potential in this animal model (*20*,*47*).

Our results with MERS-CoV C/KSA/13 suggest there might be a potential tradeoff between environmental surface stability and replication kinetics. This tradeoff was observed for a camel-derived isolate, and we did not observe similar phenotypic relationships for the other strains tested (Figures 2, 3). Future research efforts with camel-derived viruses and more closely related human-derived viruses could show whether adaptations are likely to occur after zoonosis. Our previous viral stability results with SARS-CoV-2 and the findings of this study with MERS-CoV suggest copper should be incorporated more in hospital settings, particularly in materials in areas of high contact between hospital workers and MERS patients, such as door handles, bed rails, and medical tools (*21*). Overall, we observed a range of stability, replication, and pathogenesis phenotypes between different MERS-CoV isolates, underscoring the need for continued surveillance of this virus and other coronaviruses, including SARS-CoV-2.

Appendix Additional information on surface‒aerosol stability and pathogenicity of diverse Middle East respiratory syndrome coronavirus strains, 2012‒2018.
