# Supplementary material for: Surface‒Aerosol Stability and Pathogenicity of Diverse Middle East Respiratory Syndrome Coronavirus Strains, 2012‒2018
Source: Emerg Infect Dis. 2021 Dec;27(12):3052–62. doi: 10.3201/eid2712.210344 (PMC8632154; doi:10.3201/eid2712.210344)
Supplement: Appendix — Additional information on surface‒aerosol stability and pathogenicity of diverse Middle East respiratory syndrome coronavirus strains, 2012‒2018. [file 21-0344-Techapp-s1.pdf]

Article DOI: https://doi.org/10.3201/eid2712.210344

Surface–Aerosol Stability and Pathogenicity of Diverse Middle East Respiratory Syndrome Coronavirus Strains, 2012–2018

Appendix

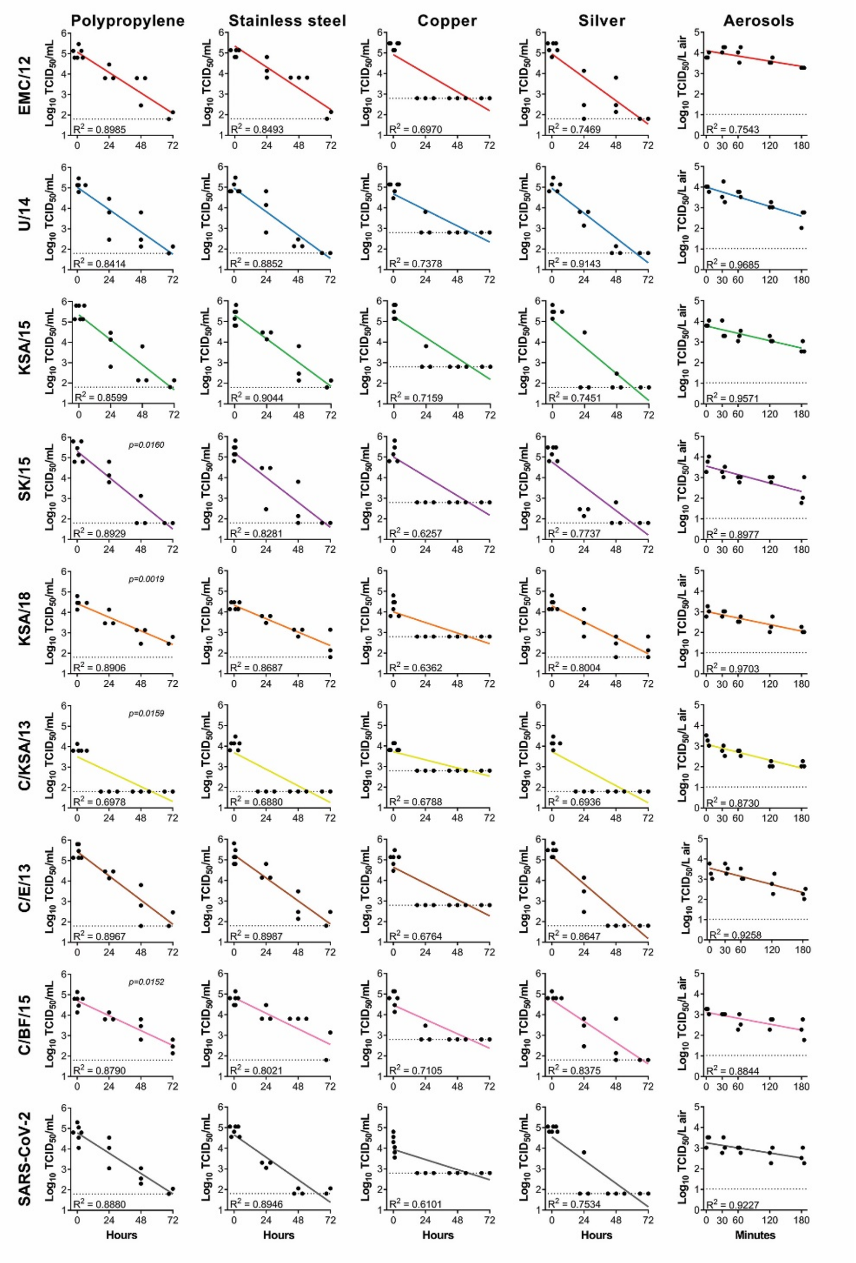

Appendix Figure. Linear regression of each stability condition for each virus. Statistically significant differences were calculated by automatic comparison of slopes by using the GraphPad (https://www.graphpad.com) simple linear regression function, followed by ordinary 1-way analysis of variance comparing EMC/12 to other strains, if differences were found. Statistically significant differences are indicated.
